# Comparison of the effects of long-lasting static stretching and hypertrophy training on maximal strength, muscle thickness and flexibility in the plantar flexors

**DOI:** 10.1007/s00421-023-05184-6

**Published:** 2023-04-08

**Authors:** Konstantin Warneke, Klaus Wirth, Michael Keiner, Lars H. Lohmann, Martin Hillebrecht, Anna Brinkmann, Tim Wohlann, Stephan Schiemann

**Affiliations:** 1 Institute for Exercise, Sport and Health, Leuphana University, 21335 Lüneburg, Germany; 2 University of Applied Sciences Wiener Neustadt, Wiener Neustadt, Austria; 3 Department of Sport Science, German University of Health and Sport, 85737 Ismaning, Germany; 4 Institute of Sports Science, Carl von Ossietzky University of Oldenburg, 26129 Oldenburg, Germany; 5 University Sports Center, Carl von Ossietzky University of Oldenburg, 26129 Oldenburg, Germany; 6 Assistive Systems and Medical Device Technology, Carl von Ossietzky University of Oldenburg, 26129 Oldenburg, Germany

**Keywords:** Maximum strength, Resistance training, Mechanical tension, Range of motion, Calf muscle

## Abstract

Maximal strength measured via maximal voluntary contraction is known as a key factor in competitive sports performance as well as injury risk reduction and rehabilitation. Maximal strength and hypertrophy are commonly trained by performing resistance training programs. However, literature shows that long-term, long-lasting static stretching interventions can also produce significant improvements in maximal voluntary contraction. The aim of this study is to compare increases in maximal voluntary contraction, muscle thickness and flexibility after 6 weeks of stretch training and conventional hypertrophy training. Sixty-nine (69) active participants (*f* = 30, *m* = 39; age 27.4 ± 4.4 years, height 175.8 ± 2.1 cm, and weight 79.5 ± 5.9 kg) were divided into three groups: IG1 stretched the plantar flexors continuously for one hour per day, IG2 performed hypertrophy training for the plantar flexors (5 × 10–12 reps, three days per week), while CG did not undergo any intervention. Maximal voluntary contraction, muscle thickness, pennation angle and flexibility were the dependent variables. The results of a series of two-way ANOVAs show significant interaction effects (*p* < 0.05) for maximal voluntary contraction (*ƞ*^2^ = 0.143–0.32, *p* < 0.006), muscle thickness (*ƞ*^2^ = 0.11–0.14, *p* < 0.021), pennation angle (*ƞ*^2^ = 0.002–0.08, *p* = 0.077–0.625) and flexibility (*ƞ*^2^ = 0.089–0.21, *p* < 0.046) for both the stretch and hypertrophy training group without significant differences (*p* = 0.37–0.99, *d* = 0.03–0.4) between both intervention groups. Thus, it can be hypothesized that mechanical tension plays a crucial role in improving maximal voluntary contraction and muscle thickness irrespective whether long-lasting stretching or hypertrophy training is used. Results show that for the calf muscle, the use of long-lasting stretching interventions can be deemed an alternative to conventional resistance training if the aim is to increase maximal voluntary contraction, muscle thickness and flexibility. However, the practical application seems to be strongly limited as a weekly stretching duration of up to 7 h a week is opposed by 3 × 15 min of common resistance training.

**Supplementary Information:**

The online version contains supplementary material available at 10.1007/s00421-023-05184-6.

## Introduction

While stretch training in humans is commonly used to improve flexibility, a meta-analysis of animal studies showed significant hypertrophic effects (Warneke et al. 2022b) with increases in muscle cross-sectional area of up to 141.6% with *d* = 5.85 as well as an increase in maximal strength of up to 95% with *d* = 12.34 following chronic stretching for six weeks. However, evidence for stretch-mediated hypertrophy and strength increases in humans is contradictory and scarce. On the one hand, Nunes et al. (2020) reviewed current literature pointing out that mostly used stretching durations in humans of up to two min per session seem not to be sufficient to induce hypertrophy. This might be explained by large differences regarding training durations (two min per session vs. chronic 24 h of stretch) as well as muscle protein synthesis between animals and humans (Garibotto et al. 1997; Sayegh and Lajtha 1989). On the other hand, there are conflicting results regarding stretch-induced maximal strength increases in humans probably based on high heterogeneity between studies regarding the way in which the stretch was induced combined with a lack in stating stretching intensity. While some studies showed significant increases in maximal strength in response to long-term stretching interventions of up to 30 min per training session (Mizuno 2019; Yahata et al. 2021), others were not able to induce significant changes in strength capacity following stretching interventions (Nakamura et al. 2021; Sato et al. 2020). All listed studies were performed including participants with a low training status or even with untrained participants. Since in animal model stretching durations of up to 24 h per day were used (Warneke et al. 2022b), a comparison to human studies performed previously seems not to be adequate. Thus, it could be assumed that previous studies in humans may not have used sufficient stretching volume (stretching duration × training frequency per week) or intensity leading to inconsistent significant increases (Nakamura et al. 2021; Nunes et al. 2020; Yahata et al. 2021). Based on this, Warneke et al. (2022a, d) investigated the effects of long-lasting static stretching interventions of up to two hours per day on seven days per week in the plantar flexors of physically active humans to improve comparability to stretching durations used in animal studies. The authors determined significant maximal strength improvements of up to 22% while—in a different study—significant stretch-mediated hypertrophy of approximately 15.3% (*d* = 0.84) could be induced by using long-lasting static stretch training of one hour per day, seven days a week (Warneke et al. 2022a, d). To date, increases in maximal strength and muscle thickness are commonly associated with resistance training routines (Ralston et al. 2017; Refalo et al. 2021; Schoenfeld et al. 2017). Different authors found maximal strength increases of 11.9% (*d* = 0.47) up to 17.0 ± 8.75% (*d* = 1.0) (Green and Gabriel 2018) as well as hypertrophic effects via magnetic resonance imaging of up to 5.2 ± 2.7% (*d* = 0.3) in young, recreationally active to moderately trained participants in the lower extremities within six weeks (Souza et al. 2014). To achieve improvements in maximal strength, on the one hand, inducing metabolic stress (Millender et al. 2021) via high training volume and frequency (Grgic et al. 2018; Ralston et al. 2017) seems to be beneficial. On the other hand, intensity regulated by mechanical loading seems to be of crucial importance to achieve maximal strength increases and hypertrophy (Krzysztofik et al. 2019; Schoenfeld et al. 2015). In resistance training, the morphological and functional adaptations are accompanied by stimulation of anabolic signaling pathways such as mTOR/p70S6k (Lamas et al. 2010; Vissing et al. 2013). Interestingly, Sasai et al. (2010) as well as Tatsumi (2010) showed the activation of this pathway due to muscle stretching. Based on very similar adaptations and underlying physiological responses, the question arises whether long-lasting stretch training could be used as an alternative to commonly used resistance training to induce significant increases in maximal strength and muscle thickness.

Consequently, the aim of the present study was to investigate the effects of long-lasting stretching interventions on maximal strength, muscle thickness and the pennation angle and compare the effects with a commonly used hypertrophy training program for the calf muscle. Since enhanced flexibility can be assumed when performing stretch training (Medeiros et al. 2016) and literature leads to the assumption that a resistance training using full range of motion (ROM) could also lead to improvements in flexibility (Afonso et al. 2021), the effects on ROM of both training interventions will be investigated as done by Warneke et al. (2022a, d). It was hypothesized that both interventions, daily long-lasting stretching and a commonly used resistance training to achieve hypertrophy, would lead to significant increases in maximal strength, hypertrophy and flexibility gains, independent of the respective intervention group.

## Methods

To compare the effects of a one-hour daily stretch training with those of a commonly used hypertrophy training, recreationally active participants were recruited from the university sports program. They were divided into a stretch training group (IG1) and a hypertrophy training group (IG2) performing either a daily long-lasting stretch training or a resistance training routine which is commonly used to induce hypertrophy in the plantar flexors. Therefore, a pre–post-design with a six-week intervention period, incorporating two maximal strength tests with extended and flexed knee joint for the plantar flexors, two flexibility tests for the range of motion in dorsiflexion of the ankle joint as well as a sonography assessment to examine changes in muscle thickness and the pennation angle were performed. Before testing, a warm-up routine consisting of five minutes of bodyweight ergometer cycling with 1 Watt/kg was performed.

### Participants

Ad hoc sample size calculation was performed using *d* = 0.7 for *F*-tests with repeated measures and within–between interaction, based on previous studies (Warneke et al. 2022a) pointing out a total sample size of at least 36 participants (12 per group). To increase the power of the investigation and counteract potential dropouts 69 recreationally active and non-competitive participants from sports study programs and local sports clubs were recruited. Participants were classified as novice to recreationally active when they performed either two or more training sessions per week in a gym or a team sport in addition to their physical education classes if they were physical education students or completing at least three resistance training sessions continuously for the previous six months. Therefore, participants had some training experience in resistance training with commonly used intensity and volume to induce hypertrophy (5 × 10–12 repetitions) as well as in team sports, such as soccer, basketball, tennis or handball. Participants with an increased risk for thromboses or serious injury in the lower extremities entailing surgery and immobilization within the past year were excluded from the study. Consequently, training status was classified as moderately trained as no untrained participants as well as no elite sport athletes were included. The participants were randomly allocated to the three groups (IG1, IG2 and CG). If participants had skipped more than three stretch training sessions or more than two resistance training sessions, respectively, data would not have been considered for further evaluation. This was, however, not the case. All participants were instructed to continue performing their previous training routines to avoid a decrease in performance in any group by stopping training. Therefore, the stretching and hypertrophy training intervention was accompanied by either the university sports program or the training routine in the gym the participants were used to. This was also the case in the control group. Characteristics of the participants are shown in Table 1.

**Table 1:** Characteristics of participants for overall sample size and divided into IG1, IG2 and CG

Group	*N*	Age (in years)	Height (in cm)	Weight (in kg)
Total	69 (*f* = 30, *m* = 39)	27.4 ± 4.4	175.8 ± 2.1	79.45 ± 5.9
IG1	23 (*f* = 10, *m* = 14)	27.4 ± 3.1	176.2 ± 5.6	81.0 ± 6.2
IG2	23 (*f* = 9, *m* = 13)	26.3 ± 2.6	175.6 ± 4.9	79.3 ± 5.3
CG	23 (*f* = 11, *m* = 12)	27.9 ± 6.1	174.4 ± 6.3	79.1 ± 7.0

*IG1* stretching group, *IG2* hypertrophy group, *CG* control group

All participants were informed about the experimental risks and provided written informed consent to participate in the present study. Furthermore, approval for this study was obtained from the university’s institutional review board (Carl von Ossietzky University of Oldenburg, No. 121-2021). The study was performed in accordance with the Helsinki Declaration.

### Testing procedure

**Fig. 1:**
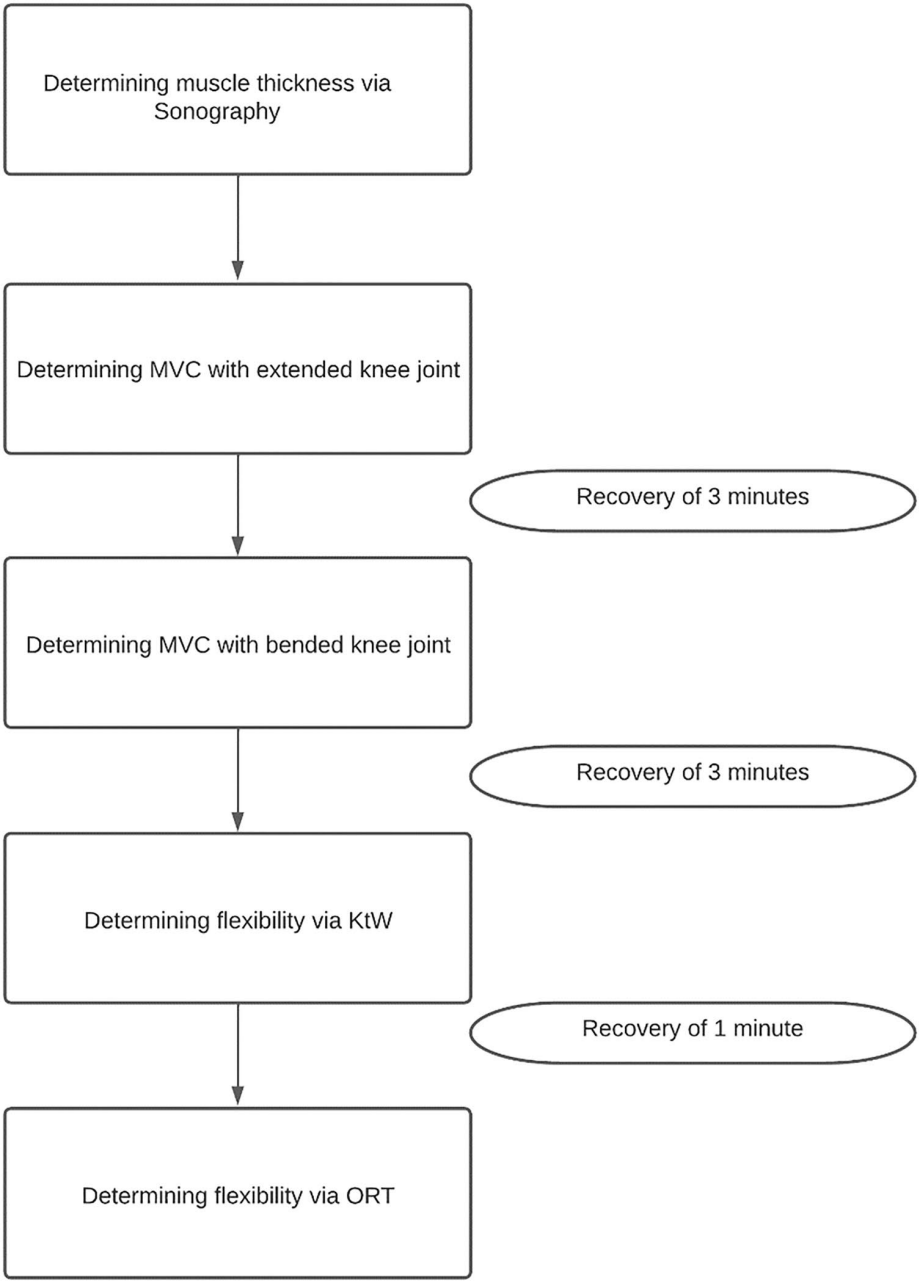
Flow chart of the testing procedure used in pre- and post-test

Figure 1 illustrates the measuring procedure used in both the pre- and post-test. The study was conducted from March to August 2022. The post-test was performed at the same time of day as the pre-test. All testing sessions were performed between 9 am and 5 pm. Participants were instructed to eat a meal latest two hours before testing.

#### Maximal strength testing

It can be assumed that there are differences in muscle innervation of the triceps surae dependent on the knee joint angle (Warneke et al. 2022c). Thus, the isometric maximal voluntary contraction was assessed using single-leg testing with extended and flexed knee joint.

#### Maximal isometric strength testing with extended knee joint

**Fig. 2:**
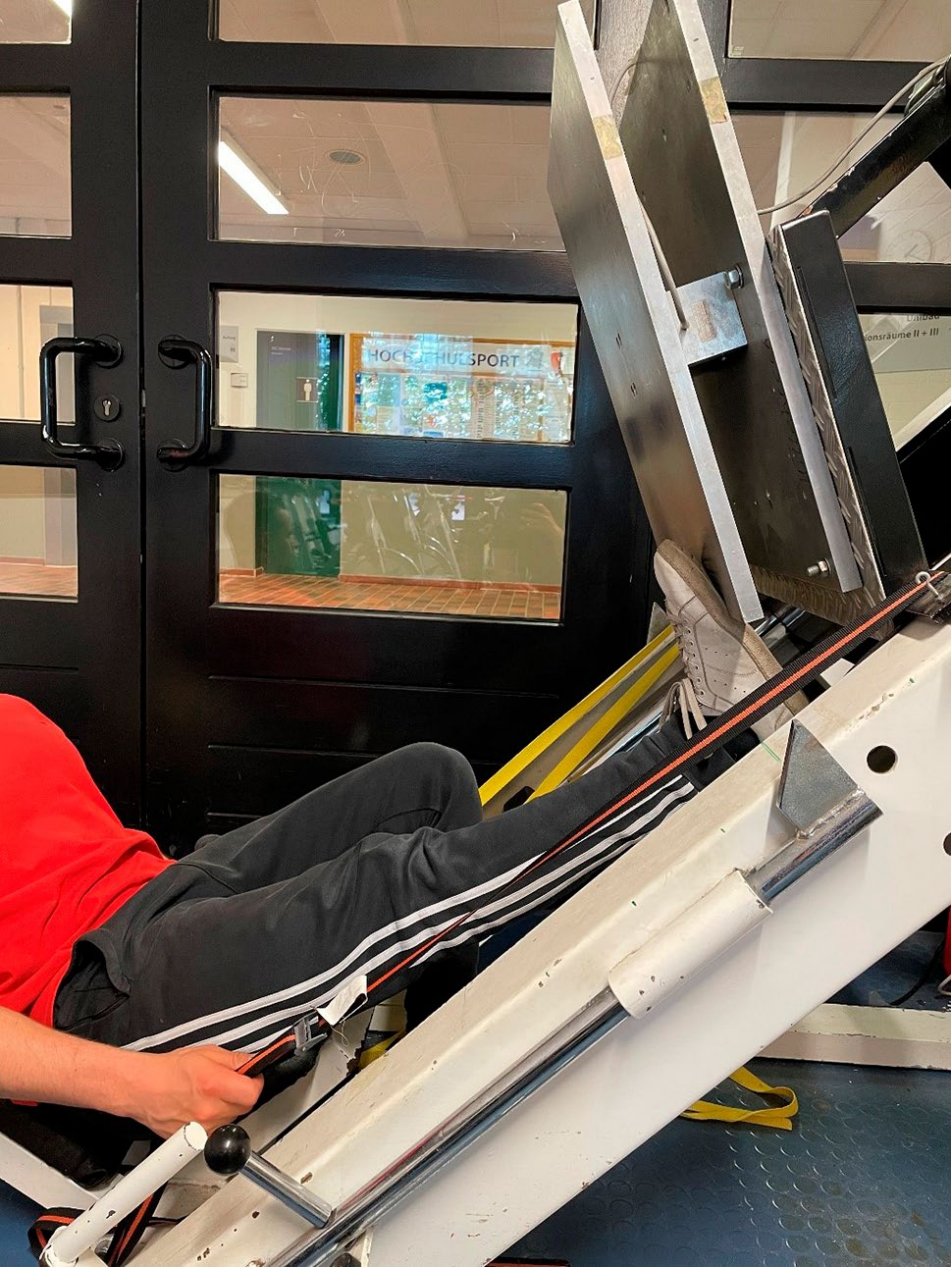
Leg press testing device for maximal isometric strength with extended knee joint (MVC180)

A 50 × 60 cm force plate with ± 5000N and a 13-bit analog-to-digital converter attached to a 45° leg press was used to measure the maximal isometric force production with an extended knee joint. In the starting position (see Fig. 2) the ankle joint angle was set to be 90°. The participants were instructed to perform a maximal plantar flexion in response to an acoustic signal and hold the maximal voluntary contraction for three seconds. After each trial, participants rested for one minute to avoid fatigue. Measurements were conducted until no improvement in maximal strength was recorded with a minimum of three trials. For isometric strength measurements, high reliability (intraclass correlation coefficient = 0.99) can be assumed (Warneke et al. 2022a).

#### Maximal isometric strength testing with flexed knee joint

**Fig. 3:**
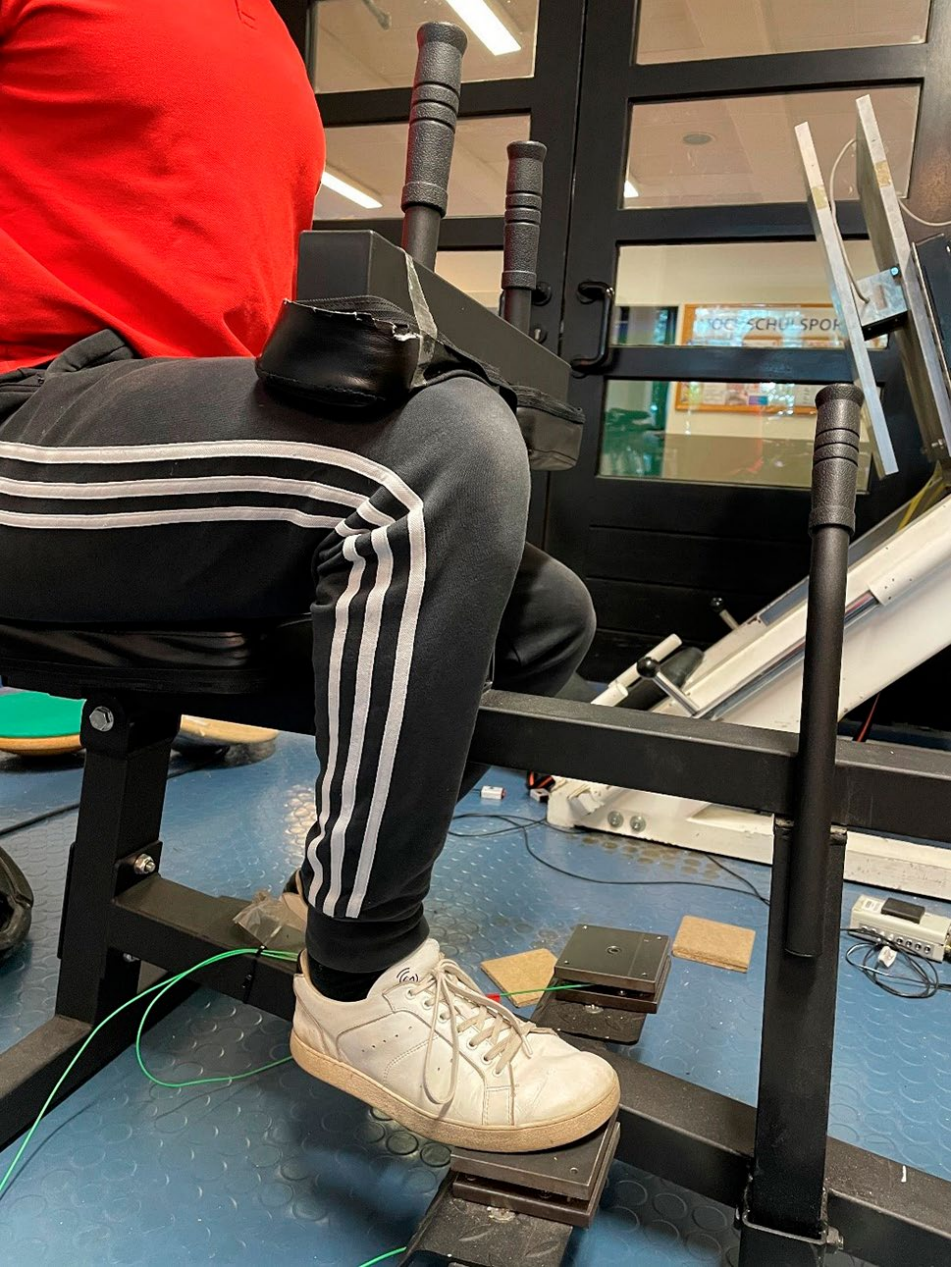
Calf muscle testing device equipped with force plates to measure maximal isometric strength with flexed knee joint (MVC90)

A calf muscle testing device was used to assess maximal isometric strength with a flexed knee joint. The maximal strength was determined using a 10 × 10 cm force plate with force sensors “Kistler Element 9251” with a resolution of 1.25N, a pull-in frequency of 1000 Hertz and a measurement range of ± 5000N. The vertical forces (Fz) were recorded via a charge amplifier “Typ5009 Charge Amplifier” and a 13-bit analog-to-digital converter NI6009 (see Fig. 3). The participants were instructed to perform maximal plantar flexion for three seconds in response to an acoustic signal. Testing was performed until participants could not improve the achieved maximal strength values with a minimum of three trials. High reliability can be assumed using maximal isometric strength testing (intraclass correlation coefficient = 0.99) (Warneke et al. 2022d).

### Determination of skeletal muscle architecture

**Fig. 4:**
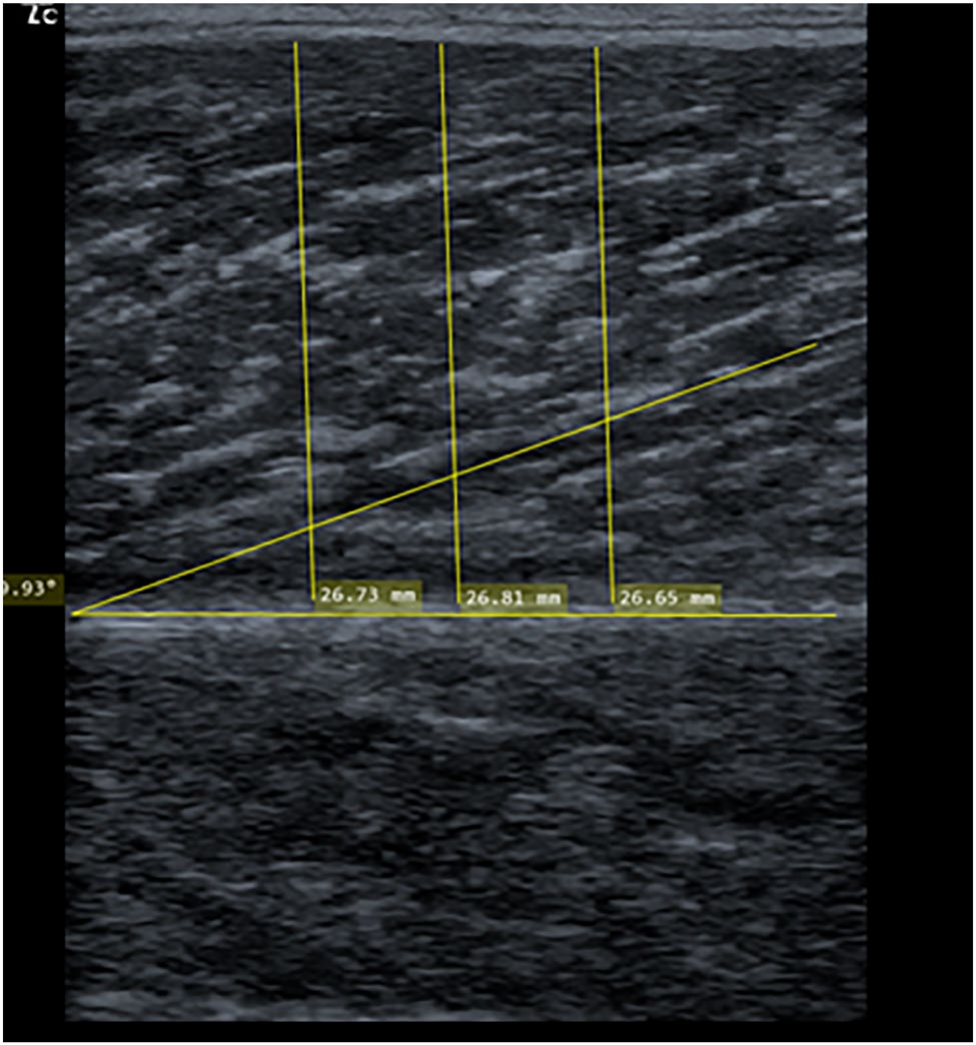
Sonography to investigate muscle thickness and pennation angle in the calf muscle

Muscle thickness and pennation angle were measured in the lateral and medial gastrocnemius using two-dimensional B-mode ultrasound with a linear transducer (12–13 MHz, Mindray Diagnostic Ultrasound System). The measurement was conducted with the participant laying in a prone position with fully extended legs and their feet hanging down at the end of a table to ensure no contraction in the calf muscles. The transducer was placed at 25% of the distance between the most lateral point of the joint space of the knee and the most lateral tip of the lateral malleolus (Perkisas et al. 2021). By holding and rotating the transducer around the sagittal-transverse axis, it was ensured that the superficial and deep aponeuroses were as parallel as possible to optimize the visibility of the fascicles as continuous striations from one aponeurosis to the other (see Fig. 4). The transducer was positioned at the midpoint of each muscle belly perpendicular to the long axis of the participant’s leg (Sarto et al. 2021). Both muscle thickness and pennation angle were obtained by averaging three measurements across the proximal, central and distal portions of the acquired ultrasound images (Franchi et al. 2017; Sarto et al. 2021). Two investigators performed the image processing independently using MicroDicom (Sofia, Bulgaria). With the measurement device stated above, the reliability can be classified as high with an intraclass correlation of 0.88–0.95 (Warneke et al. 2022a).

#### Range of motion measurement

Range of motion in the upper ankle joint was recorded via the knee-to-wall test and the goniometer on the orthosis.

#### Range of motion testing via knee-to-wall test

A sliding device was used for the knee-to-wall stretch. Participants were instructed to place the foot on the attached marker. The contralateral leg was held in the air and participants were allowed to stabilize the body with their hands placed on a doorframe. The participants pushed the board of the sliding device forward with their knee until the heel of the standing leg started to lift off. Throughout the test, the investigator pulled on a sheet of paper placed under the heel of each participant. The measurement was stopped as soon as the sheet could be removed. The distance achieved was read off in cm from the attached measuring tape. Depending on ankle range of motion, this measurement can be seen as screening flexibility with a flexed knee joint. Three valid trials were performed per leg and the furthest distance was used for evaluation. Range of motion assessment with comparable methods can be classified as high with an intraclass correlation of 0.99 (Warneke et al. 2022a).

#### Range of motion testing via goniometer of the orthosis

Range of motion in the ankle with an extended knee joint was measured via goniometer of the orthosis. For this purpose, the foot of the participant was placed on an object with the same height as the chair. While the participants were wearing the orthosis the foot was brought into a maximally dorsiflexed position keeping the knee joint in an extended position. The right angle between the lower leg and foot is classified as neutral 0°. Each big indentation of the goniometer corresponds to an increase in dorsiflexion of 5° and each little indentation corresponds to an increase of 2.5°. Range of motion assessments in the ankle joint using a goniometer can be classified as high with an intraclass correlation coefficient of 0.99 (Warneke et al. 2022a).

### Intervention

#### Stretch training (IG1)

**Fig. 5:**
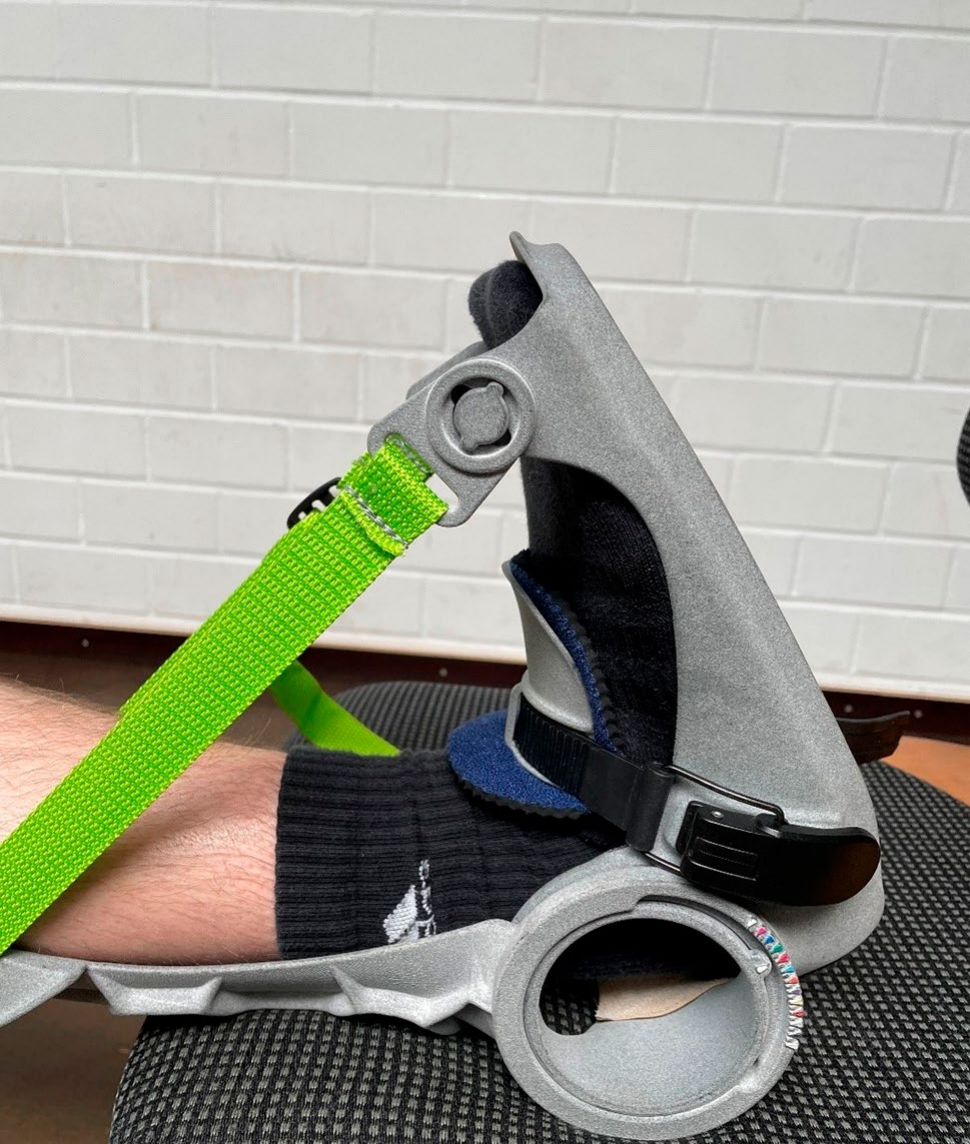
Orthosis used for calf muscle stretching

The stretching group (IG1) was instructed to perform a one hour daily stretch training for the calf muscles for six weeks. To realize this long-lasting stretch training, a calf muscle stretching orthosis was provided (see Fig. 5). The intervention was performed with the dominant leg which was determined as the leg used when performing single-leg jumps.

Subjects were instructed to wear the orthosis with an extended knee joint. To improve consistency regarding the used magnitude of stretch, the used ankle angle was quantified by the goniometer of the orthosis. Thus, the stretch could be replicated and better standardized within the six-week training intervention. Participants were instructed to reach a maximally dorsiflexed position with an individual stretching pain of 7–8 on a visual analog scale of 1–10. Participants were instructed to sit with their backs straight against the backrest and place their intervened foot on a support object at the same height as their chair. All subjects completed a stretching diary in which the daily stretching duration as well as the angle of the goniometer were written down to record the stretch duration and intensity (Fig. 5).

#### Hypertrophy training (IG2)

IG2 was instructed to perform a resistance training routine commonly used to achieve hypertrophy in the plantar flexors. Participants performed calf muscle hypertrophy training with an extended knee joint on a 45° leg press with five sets of 10–12 repetitions on three non-consecutive days per week. Training sessions lasted about 15 min. The inter-set rest was 90 s with the instruction to perform each set over full range of motion until failure. If more than 12 repetitions were accomplished, more weight was added. When a participant was not able to manage ten repetitions, the load was reduced. Participants had to complete a training diary in which training day and load were documented.

#### Statistical analysis

The analysis was performed with SPSS 28 (IBM, Armonk, New York, USA). Data is provided as mean (M) ± standard deviation (SD) for the pre–post values. The normal distribution of data was checked via Shapiro–Wilk test. Reliability was determined and is provided with intraclass correlation coefficient, coefficient of variability and 95% confidence interval (CI) for aforementioned tests (see Table 2). 95% CI for intraclass correlation coefficients and the coefficient of variability are interpreted considering the general guidelines by Koo and Li (2016): poor reliability ≤ 0.5, moderate reliability = 0.5–0.75, good reliability ≥ 0.75–0.9, excellent reliability ≥ 0.9. Reliability for sonography was determined between best and second-best value as the “within day” reliability (see Table 2). Two investigators evaluated the ultrasound images independently from one another to ensure inter-rater reliability. Moreover, Levene’s test for homogeneity in variance was performed. A one-way ANOVA was used to rule out significant differences between groups in pre-test values. A series of two-way ANOVAs with repeated measures was performed for data analyses of the pre-post comparisons. To investigate the differences in increases between the intervention groups and the control group, the Scheffé test was used as post hoc test. Effect sizes are presented as Eta squares (*ƞ*^2^) and categorized as: small effect *ƞ*^2^ < 0.06, medium effect *ƞ*^2^ = 0.06–0.14, high effect *ƞ*^2^ > 0.14 (Cohen 1988). Additionally, effect sizes are reported with Cohen’s *d* (Cohen 1988) and categorized as: small effects *d* < 0.5, medium effect *d* = 0.5–0.8, high effect *d* > 0.8. The level of significance was set to *p* < 0.05. Pearson correlations were calculated for pre–post comparisons in maximal strength and muscle thickness.

**Table 2:** Reliability for the pre-test values

Parameter	ICC (95%-CI)	CV (95%-CI) in%
MVC180	0.984 (0.978–0.989)	1.72 (1.44–2.01)
MVC90	0.983 (0.976–0.988)	1.97 (1.66–2.33)
KtW	0.991 (0.984–0.995)	0.94 (0.35–1.59)
ORT	0.992 (0.981–0.995)	0.64 (0.22–1.19)
SONOL	0.876 (0.83–0.91)	5.21 (4.4–6.15)
SONOM	0.917 (0.885–0.94)	3.5 (2.96–4.07)
PaL	0.878 (0.833–0.912)	6.64 (5.64–7.74)
PaM	0.81 (0.743–0.861)	6.49 (5.2–7.98)

*MVC* maximal voluntary contraction, *KtW* knee-to-wall test, *ORT* range of motion measurement with orthosis, *SONO* measurement of muscle thickness via sonography, *Pa* Pennation angle, *180* MVC measured with extended knee joint, *90* MVC measured with flexed knee joint, *L* lateral head of the gastrocnemius, *M* medial head of the gastrocnemius

## Results

Results of reliability are shown in Table 2. Descriptive statistics for maximal strength and flexibility are provided in Table 3 and descriptive statistics for muscle thickness and pennation angle are listed in Table 4. All data were normally distributed.

**Table 3:** Descriptive statistics and results of two-way ANOVA for maximal strength and ROM

Parameter	Pretest (M ± SD) in *N*	Post-test (M ± SD) in *N*	Pre-Post-Diff. in %	Time effect	Time × group
IG1MVC180	1522.61 ± 310.25	1796.78 ± 368.08	+ 18.00	*p* < 0.001	*p* < 0.001
IG2MVC180	1594 ± 321.78	1807.8 ± 361.11	+ 13.36	*F* = 88.26	*F* = 15.49
CG	1557.05 ± 284.46	1585.57 ± 292.04	+ 1.8	*ƞ*^2^ = 0.57	*ƞ*^2^ = 0.32
IG1MVC90	1314.7 ± 305.79	1440.61 ± 332.67	+ 9.58	*p* < 0.001	*p* = 0.006
IG2MVC90	1371.8 ± 289.45	1508.44 ± 258.7	+ 9.96	*F* = 25.908	*F* = 5.51
CG	1334.76 ± 235.36	1340.33 ± 205.81	+ 0.42	*ƞ*^2^ = 0.28	*ƞ*^2^ = 0.14
IG1KtW	11.72 ± 2.52	12.98 ± 2.55	+ 10.75	*p* < 0.001	*p* = 0.046
IG2KtW	12.26 ± 2.1	13.36 ± 2.31	+ 8.97	*F* = 48.96	*F* = 3.24
CG	11.71 ± 12.17	12.17 ± 2.0	+ 3.93	*ƞ*^2^ = 0.43	*ƞ*^2^ = 0.09
IG1ORT	8.35 ± 2.08	9.39 ± 1.41	+ 12.46	*p* < 0.001	*p* < 0.001
IG2ORT	7.92 ± 1.637	8.64 ± 1.31	+ 9.09	*F* = 39.37	*F* = 8.85
CG	8.17 ± 1.25	8.21 ± 1.03	+ 0.49	*ƞ*^2^ = 0.37	*ƞ*^2^ = 0.21

*IG1* stretching group, *IG2* hypertrophy training group, *CG* control group, *MVC* maximal voluntary contraction, *KtW* ROM Measurement via knee-to-wall test, *ORT* range of motion measurement via goniometer of the orthosis, *180* MVC testing in extended knee joint, *90* MVC testing in flexed knee joint

**Table 4:** Descriptive statistics of muscle thickness and the pennation angle

Parameter	Pretest (M ± SD) in *N*	Post-test (M ± SD) in *N*	Pre-Post-Diff. in %	Time effect	Time × group
IG1MThL	14.53 ± 2.43	15.21 ± 2.11	+ 4.68	*p* < 0.001	*p* = 0.021
IG2MThL	14.83 ± 2.91	16.09 ± 3.35	+ 8.5	*F* = 15.51	*F* = 4.08
CG	14.33 ± 2.48	14.40 ± 2.32	+ 0.49	*ƞ*^2^ = 0.19	*ƞ*^2^ = 0.11
IG1MThM	19.55 ± 2.59	21.06 ± 2.88	+ 7.72	*p* < 0.001	*p* = 0.006
IG2MThM	19.25 ± 3.47	20.87 ± 3.09	+ 8.42	*F* = 19.46	*F* = 5.48
CG	18.49 ± 3.13	18.41 ± 2.87	− 0.43	*ƞ*^2^ = 0.23	*ƞ*^2^ = 0.14
IG1PaL	13.39 ± 2.33	13.49 ± 2.73	+ 0.75	*p* = 0.549	*p* = 0.625
IG2PaL	14.14 ± 2.91	14.59 ± 2.28	+ 3.18	*F* = 0.36	*F* = 0.47
CG	12.67 ± 2.86	12.55 ± 2.76	− 0.95	*ƞ*^2^ = 0.01	*ƞ*^2^ = 0.02
IG1PaM	17.32 ± 4.07	19.46 ± 3.24	+ 12.3	*p* < 0.001	*p* = 0.077
IG2PaM	16.92 ± 3.18	19.07 ± 3.04	+ 12.71	*F* = 12.81	*F* = 2.66
CG	16.51 ± 3.92	16.62 ± 3.67	+ 0.67	*ƞ*^2^ = 0.16	*ƞ*^2^ = 0.08

*IG1* stretching group, *IG2* hypertrophy training group, *CG* control group, *MThL* muscle thickness in the lateral head of gastrocnemius, *MThM* muscle thickness in the medial head of gastrocnemius, *PaL* pennation angle in the lateral head of the gastrocnemius, *PaM* pennation angle in the medial head of the gastrocnemius

The evaluation of pre-test group differences showed no significance between groups (*F* = 0.161–1.699, *p* = 0.191–0.813).

Table 3 shows the descriptive statistics of the maximal strength and flexibility assessment in plantar flexion.

### Maximal strength analysis

**Fig. 6:**
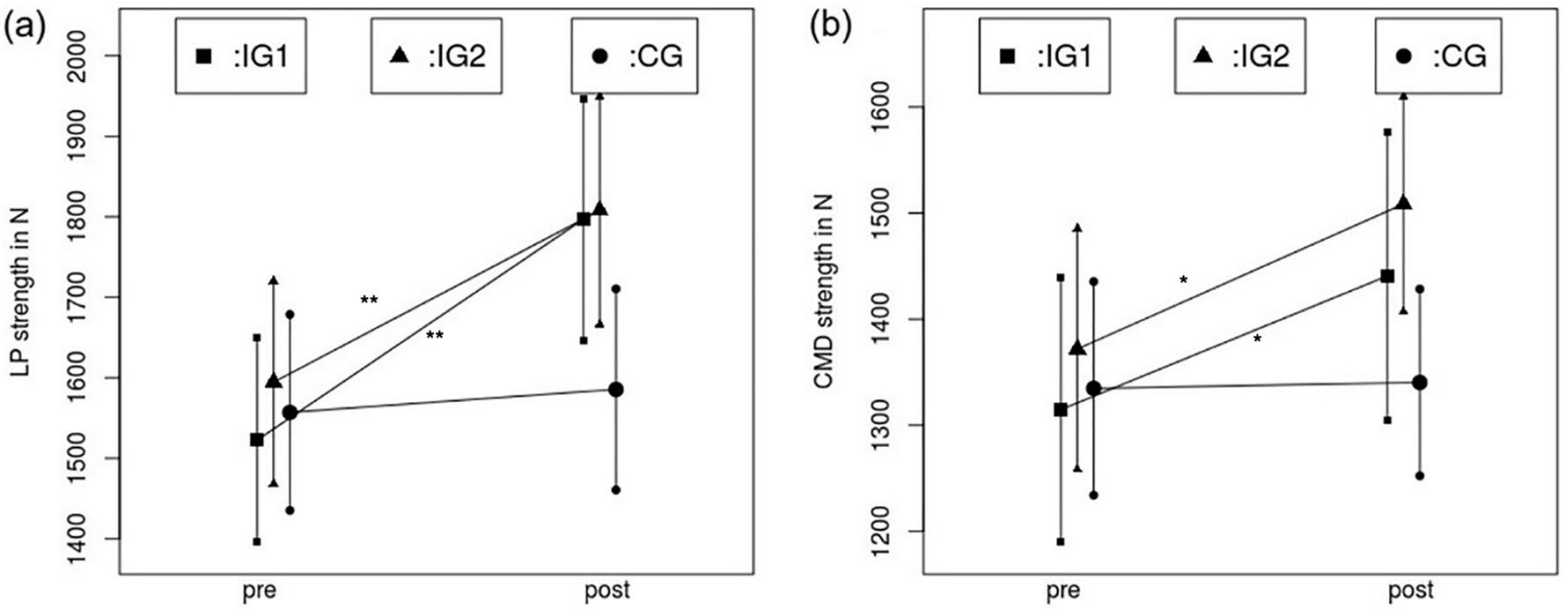
Comparison of maximal strength from pre- to post-test in the stretching group (IG1), the hypertrophy training group (IG2) and the control group (CG) with extended (**a**) and flexed knee joint (**b**). ** indicates a significant increase compared to the control group of *p* < 0.001, * indicates a significant increase compared to the control group of *p* < 0.05

Figure 6 illustrates changes in maximal strength using the maximal strength measurement with extended and flexed knee joint for the intervened leg.

#### Plantar flexor maximal voluntary contraction with extended knee joint

Results for maximal strength measured using the maximum voluntary contraction in the plantar flexors with extended knee joint showed high, significant increases with a time effect of *ƞ*^2^ = 0.572, *p* < 0.001 and a significant time × group interaction (*ƞ*^2^ = 0.319, *p* < 0.001). Post hoc testing pointed out no significant differences for increases from pre- to post-test between the stretching group (IG1) and the hypertrophy training group (IG2) (*p* = 0.387, *d* = 0.4) but differences in favor of the intervention groups between the stretching group (IG1) and the control group (CG) (*p* < 0.001, *d* = 1.17) as well as between the hypertrophy training group (IG2) and the control group (CG) (*p* < 0.001, *d* = 0.9). Therefore, no change in the control group but significant increases in both intervention groups were obtained.

#### Plantar flexor maximum voluntary contraction with flexed knee joint

Results for maximal strength in the plantar flexors measured with flexed knee joint also showed a high, significant increase with a time effect of *ƞ*^2^ = 0.282, *p* < 0.001 and a significant time × group interaction (*ƞ*^2^ = 0.143, *p* = 0.006). Furthermore, post hoc testing pointed out no significant difference for the increases in maximal strength between the stretching group (IG1) and the hypertrophy training group (IG2) (*p* = 0.986, *d* = 0.05). There were differences in favor of the intervention groups with moderate effect sizes between the stretch training group (IG1) and the control group (CG) (*p* = 0.029, *d* = 0.6) as well as between the hypertrophy training group (IG2) and the control group (CG) (*p* = 0.013, *d* = 0.651). Therefore, the results show significant increases in both intervention groups without any significant change in the control group.

### Range of motion analysis

#### Range of motion via knee-to-wall stretch

Results of the knee-to-wall test demonstrated high, significant increases with a time effect of *ƞ*^2^ = 0.426, *p* < 0.001 and a time × group interaction (*ƞ*^2^ = 0.169, *p*  = 0.046). Post hoc testing showed no significant differences between the increases of the stretching (IG1) and hypertrophy training group (IG2) with *p* = 0.882, *d* = 0.24, while there were moderate magnitudes in effect sizes for differences in favor of the intervention groups between the stretching group (IG1) and the control group (CG) (*p* = 0.062, *d* = 0.53) as well as between the hypertrophy training group (IG2) and the control group (CG) (*p* = 0.152, *d* = 0.42), showing increases in all groups without a significant difference between groups..

#### Range of motion via goniometer of the orthosis

Furthermore, there was a high, significant increase in the flexibility measured with the goniometer of the orthosis with a time effect of *ƞ*^2^ = 0.374, *p* < 0.001 and a significant, high time × group interaction (*ƞ*^2^ = 0.212, *p* < 0.001). Post hoc testing determined no significant difference for the increases between the stretching (IG1) and the hypertrophy training group (IG2) (*p* = 0.378, *d* = 0.38). There were significant differences in favor of the intervention groups between the stretching group (IG1) and the control group (CG) (*p* < 0.001, *d* = 0.9) and the hypertrophy training group (IG2) and the control group (CG) (*p* = 0.022, *d* = 0.61), showing no significant change in the control group, while there were significant range of motion increases in both intervention groups.

### Muscle thickness and pennation angle analyses

Table 4 shows the descriptive statistics for muscle thickness and the pennation angle in the lateral and medial gastrocnemius.

#### Muscle thickness in lateral and medial head of the gastrocnemius

**Fig. 7:**
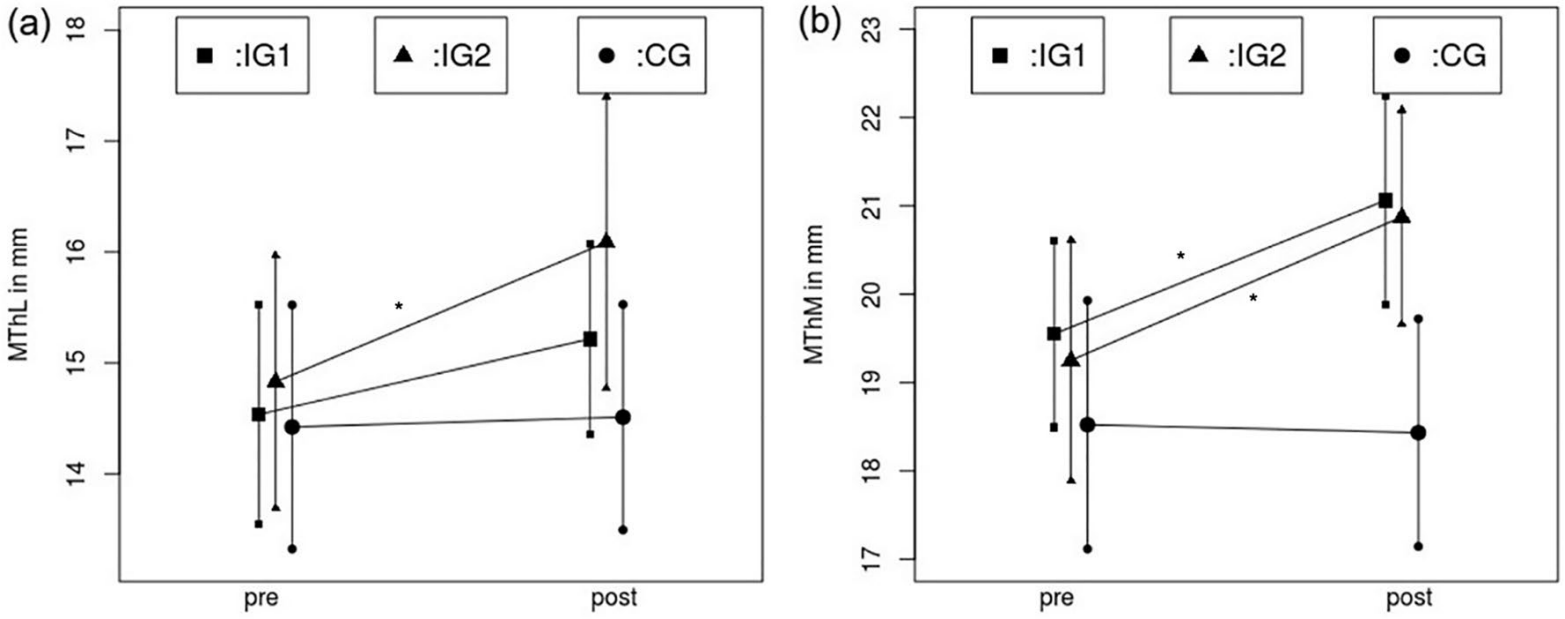
Muscle thickness comparison from pre- to post-test in the stretching group (IG1), the hypertrophy training group (IG2) and the control group (CG) in the lateral (**a**) and medial (**b**) gastrocnemius. ** indicates a significant increase compared to the control group of *p* < 0.001, * indicates a significant increase compared to the control group of *p* < 0.05

Figure 7 illustrates changes in the muscle thickness measured via sonography in the lateral and medial gastrocnemius in all three groups.

Results for muscle thickness measurement in the lateral head of the gastrocnemius showed a significant increase from pre- to post-test with a time effect of *ƞ*^2^ = 0.19, *p* < 0.001 with a moderate, significant interaction effect (time × group, *ƞ*^2^ = 0.11, *p* = 0.021). In the medial head of the gastrocnemius, there was a high, significant increase in muscle thickness showing a time effect of *ƞ*^2^ = 0.228, *p* < 0.001 with a significant time x group interaction (*ƞ*^2^ = 0.142, *p* = 0.006).

For the lateral head of the gastrocnemius, post hoc testing pointed out significant differences in favor of the intervention group (IG2) with moderate effect sizes between the hypertrophy training group and (IG2) and the control group (CG) (*p* = 0.021, *d* = 0.61) but no significant differences could be observed between the stretching group (IG1) and the control group (CG) (*p* = 0.36, *d* = 0.32) and the stretching group (IG1) and the hypertrophy training group (IG2) (*p* = 0.37, *d* = 0.32). Therefore, no significant increase was found for the stretching group compared with the control group, while there was a significantly greater increase in the muscle thickness of the lateral head in the IG2. In the medial head of the gastrocnemius, no significant difference was found between the stretching (IG1) and the hypertrophy training group (IG2) (*p* = 0.979, *d* = 0.03), however, there were significant differences in favor of the intervention groups with moderate effect sizes between the stretching group (IG1) and the control group (CG) (*p* = 0.027, *d* = 0.6) as well as between the hypertrophy training group (IG2) and the control group (CG) (*p* = 0.014, *d* = 0.646), showing significant hypertrophy in IG1 and IG2 without a difference between the groups, while no significant changes could be obtained in the control condition.

Individual progressions of the listed parameters are illustrated in separate figures in the supplemental material.

#### Pennation angle in the lateral and medial head of the gastrocnemius

For the pennation angle in the lateral head of the gastrocnemius, no significant increase from pre- to post-test could be observed (time effect of *p* = 0.549, *ƞ*^2^ = 0.006, time × group interaction *p* = 0.625, *ƞ*^2^ = 0.015). In the medial head of the gastrocnemius, there was a high, significant time effect (*p* < 0.001, *ƞ*^2^ = 0.163), however, no significant time × group interaction (*p* = 0.077, *ƞ*^2^ = 0.075) could be found.

## Discussion

The present study compared the effects of a one hour daily stretching intervention in the plantar flexors with a commonly used hypertrophy training routine over a period of six weeks. Results showed an increase in maximal strength with moderate to high effects (*ƞ*^2^ = 0.143–0.572, *d* = 0.6–1.17, *p* < 0.001–0.006), low to moderate effects for increases in muscle thickness (*ƞ*^2^ = 0.11–0.228, *d* = 0.32–0.65, *p* < 0.001–0.021) as well as low to high effects for increases in flexibility (*ƞ*^2^ = 0.089–0.426, *d* = 0.42–0.9, *p* < 0.001–0.046) irrespective of performing a commonly used hypertrophy training or long-lasting stretching for the calf muscle. The control group exhibited no significant changes in any measured value. Results showed that there was no significant difference in adaptations between the stretching and hypertrophy training group regarding increases in maximal strength, muscle thickness and flexibility (*p* = 0.37–0.99, *d* = 0.03–0.4). Therefore, performing stretch training can be assumed to provide a sufficient stimulus to increase maximal strength and hypertrophy in the calf muscle if performed with adequate training volume (stretch duration × weekly frequency), which is comparable to adaptations of commonly used resistance training.

Previous studies were able to show stretch-mediated strength increases as well. Nelson et al. (2012) and Yahata et al. (2021) pointed out improvements in maximal strength of up to 29% (*d* = 1.24) and 6.6% (*d* = 0.35) using lower stretching durations of 4 x 30 s three times per week and 30 min per session two times per week, respectively. Considering a stretch-induced increase in maximal strength of 29% by using 4 × 30 s of stretching, the included participants should be stated as untrained, as listed increases would be higher as expectable effects of resistance training programs. Since Nelson et al. (2012) described their participants as physically inactive or “minimally recreationally active” by performing training less than five times per months for less than 60 min per session, the training level of the participants included in the present study must be considered as significantly higher. While Nunes et al. (2020) reviewed current literature pointing out no significant influence of stretch training on hypertrophy, the only studies that used long-lasting stretching (> 30 min of stretch per session) with a daily frequency showed significant, stretch-mediated hypertrophy and maximal strength increases (Warneke et al. 2022a), comparable with previous animal studies (Kelley 1996; Warneke et al. 2022b).

In animal studies (Frankeny et al. 1983) and also in human studies (Warneke et al. 2022a, c; Yahata et al. 2021) higher adaptations were found by increasing stretching duration and volume. Since in resistance training, previous authors pointed out increases in strength capacity of about 17.0 ± 8.75% (*d* = 1.0) (Green and Gabriel 2018; Grgic et al. 2018) and Warneke et al. (2022a) showed comparable increases in strength and muscle thickness in response to one hour of daily stretching, these long durations seem to be necessary to achieve an adequate stimulus.

It is well known that mechanical tension (intensity) plays a crucial role in physiological adaptations when aiming to induce hypertrophy but especially for maximal strength improvements, which is accompanied by a stimulation of anabolic signaling pathways (Schoenfeld et al. 2015; Wackerhage et al. 2019). Literature points out the possibility to induce similar mechanical tension and therefore anabolic signaling due to the activation of so-called stretch-activated channels (Suzuki and Takeda 2011), resulting in stimulating mTOR signaling pathways (Tyganov et al. 2019). Therefore, increases in maximal strength are possibly explained by mechanical tension-induced adaptations which one could speculate to be similar to adaptations of a common hypertrophy training, including increases in muscle quality, muscle thickness and architecture and/or elongation of the muscle-tendon unit. Accordingly, in animal studies, Devol et al. (1991) referred to mechanical tension per sarcomere as an important factor to induce stretch-related responses in the muscle, however, in humans the underlying physiological processes of stretch-activated increases in maximal strength and muscle thickness remain unclear. A previous study (Warneke et al. 2022a) found no relationship between increases in muscle thickness and maximal strength.

Noticeable, even though there are several similarities regarding the adaptations over the six-week period following stretching and hypertrophy training in the results reported in this study (regarding maximal strength, muscle thickness and flexibility), it can be assumed that resistance training would lead to further health-related benefits, such as improved cardiovascular function (Schjerve et al. 2008; Yu et al. 2016) and bone mineral density (Westcott 2012). To this point, it remains unclear whether and to which extend long-lasting stretching would be effective concerning health-related parameters.

It is well known that neuronal factors play an essential role in maximal strength increases in the first weeks of training (Del Vecchio et al. 2019) while structural adaptations might play a secondary role (Gabriel et al. 2006). Consequently, it can be assumed that enhanced strength capacity could be primally explained by neuronal changes. The potential neuromuscular adaptations leading to stretch-mediated increases in maximal strength capacity still remain unclear. Holly et al. (1980) pointed out that no significant increase in central nervous activity was found when inducing long-term stretching in animal models, while Sola et al. (1973) pointed out significant stretch-mediated hypertrophy even if the muscle was previously denervated. Therefore, further investigations are requested to clarify the physiological mechanism of stretch-induced maximal strength increases. In contrast, benefits of central nervous innervated muscle contraction such as motor learning effects can be hypothesized to occur in a lower magnitude compared to active training protocols.

However, even though transferability of results from animal research should be considered carefully, in animal model the morphological adaptations are investigated more frequently, pointing out a serial accumulation of sarcomeres in response to chronic stretching interventions even after a few days (Antonio et al. 1993) which could also be responsible for increased muscle mass and, due to optimizing the length–tension relationship, for changes in force production capability of the muscle. Hypothesizing a general transferability to humans, these adaptations could also indicate changes in muscle morphology which could contribute to significant maximal strength increases. Furthermore, since an increased muscle thickness was measured, an enhancement in the pennation angle was reasonably hypothesized (Cormie et al. 2011). Accordingly, the pennation angle seems to increase with enhancement in muscle thickness in both groups. This may also be responsible for improvements in maximal strength as an increase in the number of contractile filaments in parallel and a higher strength capacity can be assumed (Cormie et al. 2011).

However, even without a significant difference between the stretching group (IG1) and the hypertrophy training group (IG2) the comparatively high time-effort of the stretch training should be considered, as the time spent with training for IG1 was long compared with IG2. While IG2 performed their training routine within a weekly duration of about 45 min (3 × 15 min), IG1 had to stretch the plantar flexors for up to seven hours per week. Furthermore, the stretching group performed their training routine more frequently (seven days per week) than the hypertrophy training group (three times per week). Even with (non-significant) higher increases in maximal strength in the stretching group, the time-effort of this group can be assumed to be unproportionally high compared with the hypertrophy training group. However, the training of IG1 could be integrated in the daily life or prolonged times of immobilization, which was not possible for IG2, as the hypertrophy orientated training protocol required a leg press machine.

It is well accepted that performing stretch training results in improved flexibility (Medeiros and Lima 2017). There are many hypotheses trying to explain increases in range of motion after a stretch training. While authors hypothesize an increased tolerance of stretching tension via a reduced pain sensitivity (Freitas et al. 2018), animal models show evidence of structural adaptations by a serial accumulation of sarcomeres (Antonio and Gonyea 1993). However, when resistance training is performed over full range of motion, improvements in range of motion can be assumed as well (Afonso et al. 2021). There are many theories explaining the increases in muscle flexibility and joint range of motion, pointing out neuromuscular changes (Freitas et al. 2018; Freitas and Mil-Homens 2015) and structural changes in the muscle–tendon unit and reduction in passive peak torque (Moltubakk et al. 2021; Nakamura et al. 2017). The described increased number of serial sarcomeres in animals (Antonio et al. 1993; Warneke et al. 2022b)was, to the best knowledge, not confirmed in humans.

In the supplemental material, the individual progressions were reported for the significant results of this study, showing no difference in consistency of the increment of maximal strength between stretch-mediated hypertrophy and resistance training-induced hypertrophy as well as maximal strength increases (Suppl. Fig. A–F). Since most previous studies were performed with untrained participants, this study was conducted with (recreationally) active participants, showing a comparatively wide range of strength and flexibility level as well as in muscle thickness. Although lower adaptations can be assumed in trained participants, the stretch-mediated hypertrophy was also effective in participants with higher strength levels and/or muscle thickness. However, since the study was conducted over a period of only six weeks, investigations using longer training durations are requested to exclude strong adaptations because of an unfamiliar training stimulus.

### Limitations

Since testing of maximal strength was performed under isometric conditions, higher increases in the stretching group might be explained with contraction-specificity because of proximity to the intervention stimulus (Lanza et al. 2019). To improve comparability to dynamic conditions, dynamic one repetition maximum testing should be included in future testing as hypertrophy training of IG2 was performed dynamically but tested under isometric conditions. There is limited transferability of isometric strength to one repetion maximum measurements (Murphy and Wilson 1996). In contrast to maximal strength increases, there was higher hypertrophy in the gastrocnemius in the resistance training compared to the stretching group. This may be explained due to the use of different joint angles and, therefore, used stimuli in different muscle length while stretching used maximal range of motion only. In both groups, the interventions seem to be more effective for increases in muscle thickness of the medial head of the gastrocnemius. To rule out adaptations based on an unfamiliar stimulus or only adaptations in the first phase of training, investigations examining longer intervention periods are requested. As this study compared the effects of a one hour daily stretching routine to the effects of a hypertrophy training using 5 × 10-12 repetitions performed three times per week, obviously, the time under tension as well as the intensities cannot be compared with one another. However, this was not the aim of this study as the effects of two different training routines are contrasted. Furthermore, inconsistency in the wording to describe the training status of included participants throughout the studies should be considered when interpretating the results of these studies. No statement can be given about the effects in highly trained participants, as no previous research investigated long-lasting stretching in elite athletes.

Furthermore, ultrasound imaging to investigate hypertrophy following training interventions seems to be biased by limited objectivity and a lack of accuracy (Warneke et al. 2022e). Therefore, using magnetic resonance imaging measurements to confirm morphological adaptations should be considered in future study designs.

In general, there is no “real” quantification of stretching intensity in many studies in humans. Using stretching pain as an indicator for stretch intensity seems to be biased, as Lim and Park (2017) pointed limited correlations between stretching pain and passive peak torque. Assuming mechanical tension is of crucial importance for adaptations in maximal strength and hypertrophy, the passive torque of the muscle should be considered as relevant. Therefore, no studies could be found addressing the effects of different intensities which could be of high impact for the practicability of the stretching routine, since it might be hypothesized that using higher intensities could reduce the required stretching duration to reach comparable adaptations.

Lastly, the influence of training level, sex and age was not investigated in this study. However, the sex-dependent adaptations were previously investigated by Warneke et al. 2023. To investigate further independent variables’ influence such as age and training level, a more heterogeneous group of participants should have been included to the study.

## Practical applications

Results point out long-lasting stretch training (one hour daily, high elongation stress) as a promising alternative to resistance training (e.g., hypertrophy training) in different settings over a six-week period, especially if commonly used resistance training is contraindicated, e.g., after injury and surgery. There are some advantages of long-lasting stretch training for athletes and patients to perform their training routine independent of training equipment like the leg press or calf muscle machines which are required for traditional resistance training of the plantar flexors to achieve hypertrophy.

### Outlook

Long-lasting stretching interventions produced significant hypertrophy and maximal strength gains in animal studies (Antonio et al. 1993; Bates 1993; Warneke et al. 2022b). In humans, more evidence regarding long-lasting stretching interventions and its impact on maximal strength and muscle thickness is required. Even though Nunes et al. (2020) showed that short-lasting stretching is not sufficient to induce hypertrophy, previous research shows that long-lasting stretching interventions can induce sufficient tension to improve maximal strength, range of motion and muscle thickness (Warneke et al. 2022a). The present study also showed significant increases over a six-week period in the measured parameters which are comparable to those of a commonly used resistance training in the plantar flexors. Since significant decreases in strength capacity, flexibility as well as muscle thickness due to immobilization (Stevens et al. 2004) after injury and/or surgery can be assumed, the results of this study are promising as a method with high potential in rehabilitation of orthopedic indications. Therefore, studies including clinical trials and older participants should be performed. To investigate the underlying physiological adaptations leading to increased strength capacity as well as hypertrophy, neuromuscular adaptations (for example via EMG) as well as further morphological adaptations should be addressed in further studies.

## Supplementary Information

Below is the link to the electronic supplementary material.

Supplementary file1 (DOCX 924 KB)

## Data Availability

Data can be provided by the corresponding author due to reasonable request.

## Abbreviations

MVC: Maximal voluntary contraction
ROM: Range of motion
MSt: Maximal strength
mTOR: Mammalian target of rapamycin
p70S6k: Protein S6 kinase
IG1: Intervention group 1
IG2: Intervention group 2
CG: Control group
ICC: Intraclass correlation coefficient
CMD: Calf muscle device
ORTH: Goniometer of the orthosis
KtW: Knee-to-wall test
M: Mean
SD: Standard deviation
ANOVA: Analysis of variance
MTh: Muscle thickness
LP: Leg press
