# Supplementary material for: Comparison of the effects of long-lasting static stretching and hypertrophy training on maximal strength, muscle thickness and flexibility in the plantar flexors
Source: Eur J Appl Physiol. 2023 Apr 8;123(8):1773–87. doi: 10.1007/s00421-023-05184-6 (PMC10363083; doi:10.1007/s00421-023-05184-6)
Supplement: Supplementary file 1 — Supplementary file1 (DOCX 924 KB) [file 421_2023_5184_MOESM1_ESM.docx]

**Supplemental Material**


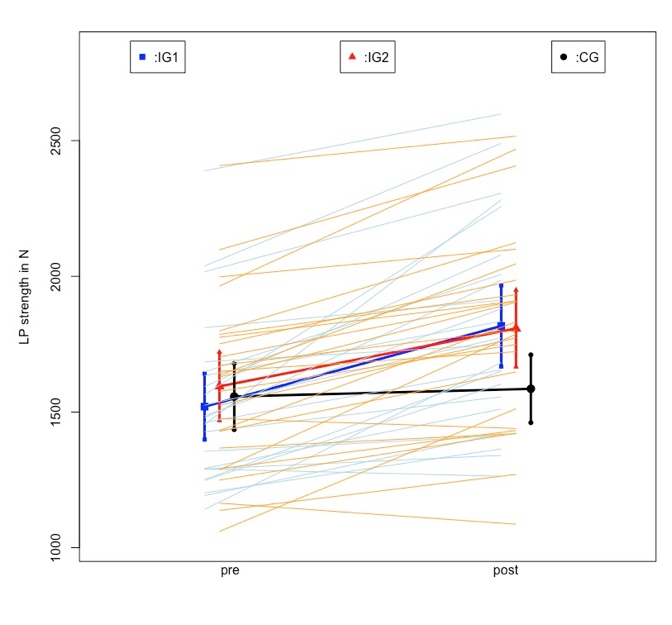

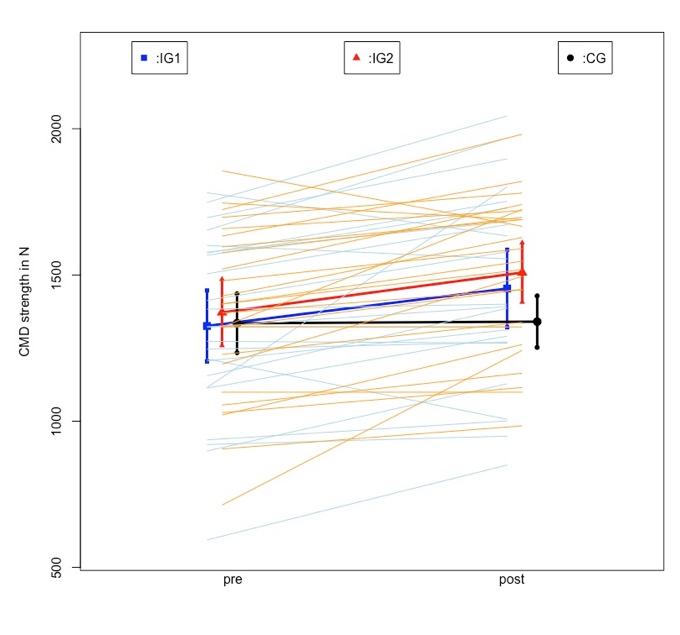


Figure A Comparison of maximal strength from pre- to post-test in the stretching group (IG1), the hypertrophy training group (IG2) and the control group (CG) with extended (a) and bent knee joint (b) under consideration of individual progressions


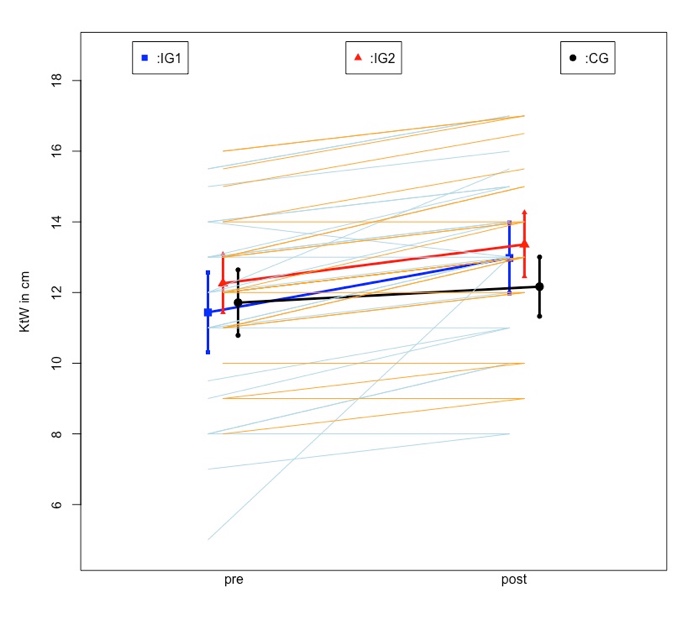

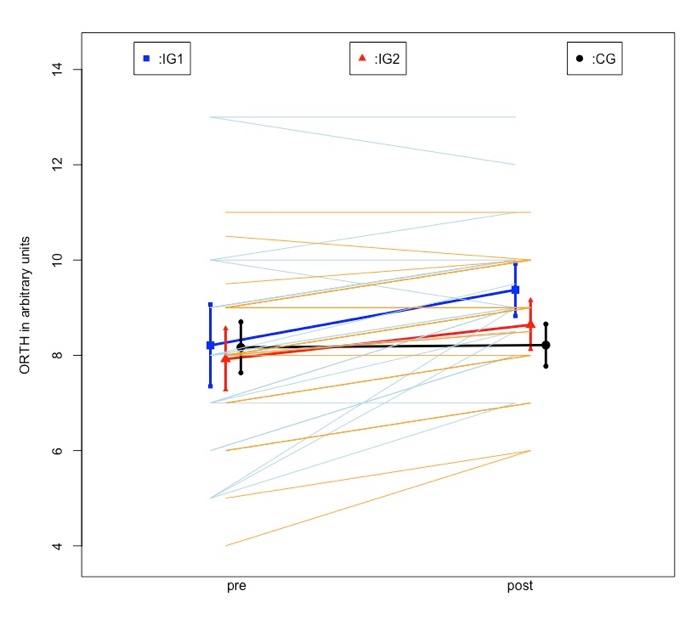


Figure B Comparison of flexibility from pre- to post-test in the stretching group (IG1), the hypertrophy training group (IG2) and the control group (CG) in the knee to wall stretch (a) and via the goniometer of the orthosis (b) under consideration of individual progressions


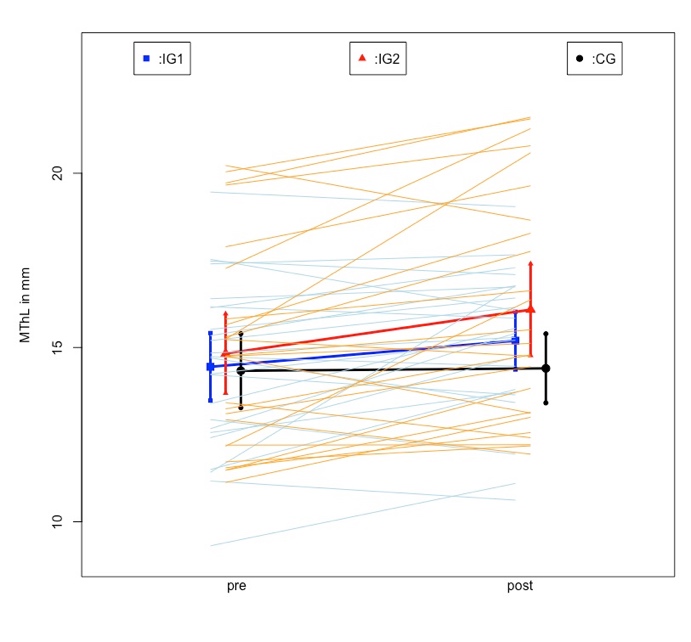

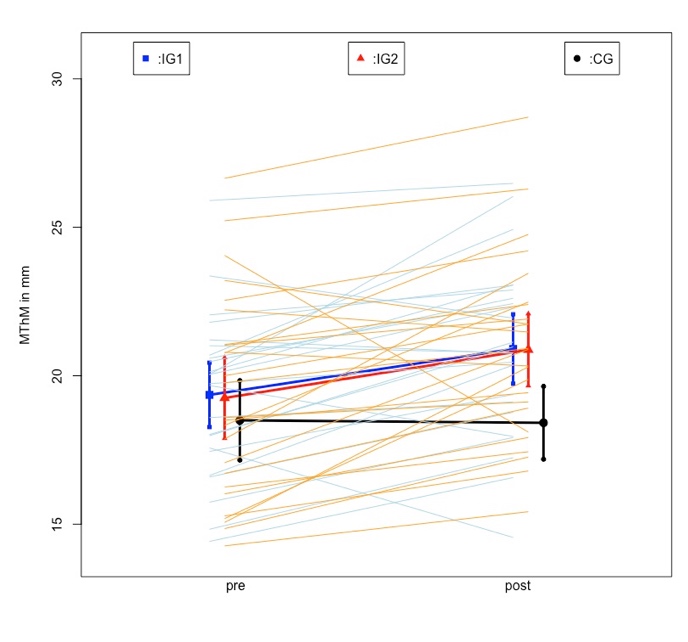


Figure C Comparison of hypertrophy from pre- to post-test in the stretching group (IG1), the hypertrophy training group (IG2) and the control group (CG) in lateral head of the gastrocnemius (a) and the medial head of the gastrocnemius (b) under consideration of individual progressions


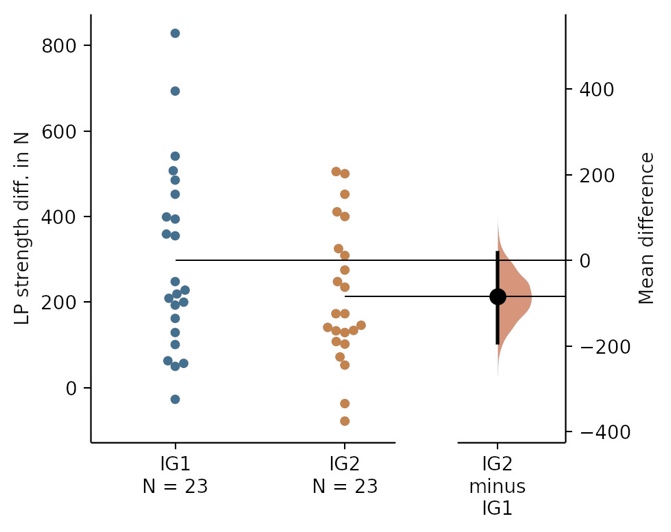

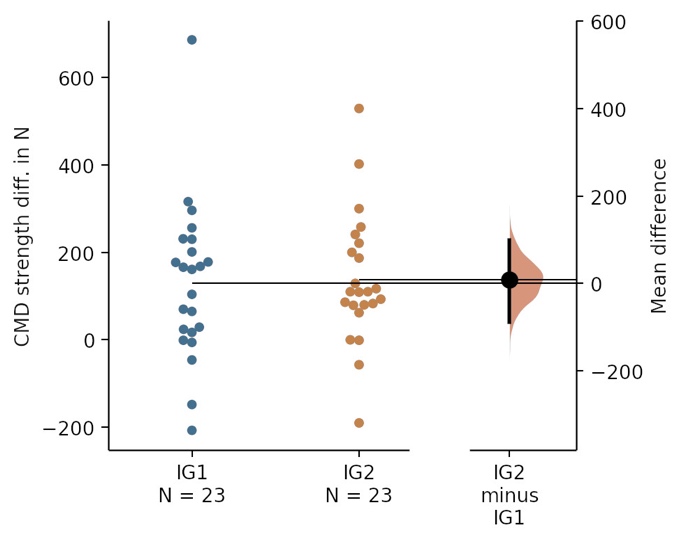


Figure D Comparison of increases in maximal strength in the stretching group (IG1)and the hypertrophy training group (IG2) with extended and bent knee joint illustrating the group differences in mean differences


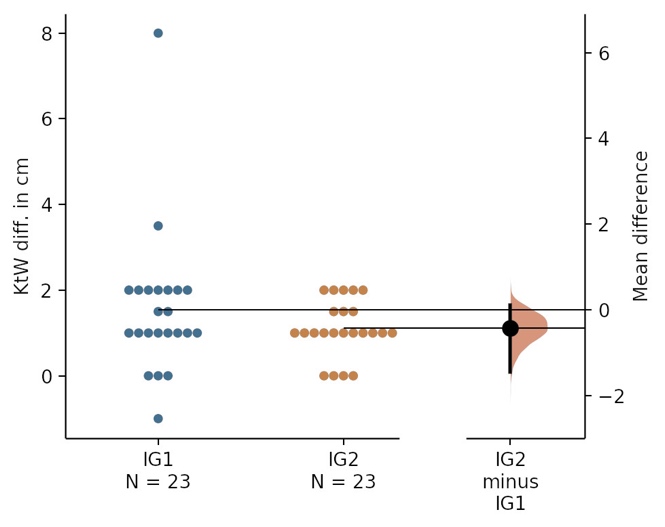

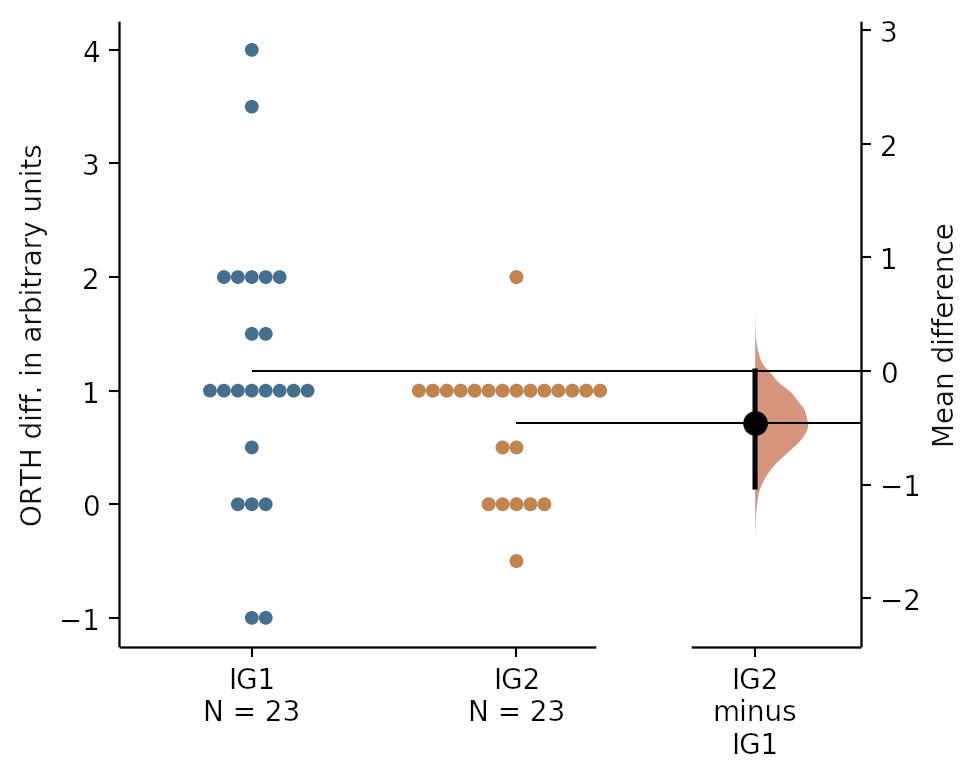


Figure E Comparison of increases in flexibility in the stretching group (IG1)and the hypertrophy training group (IG2) in the knee to wall stretch and via the goniometer of the orthosis illustrating the group differences in mean differences


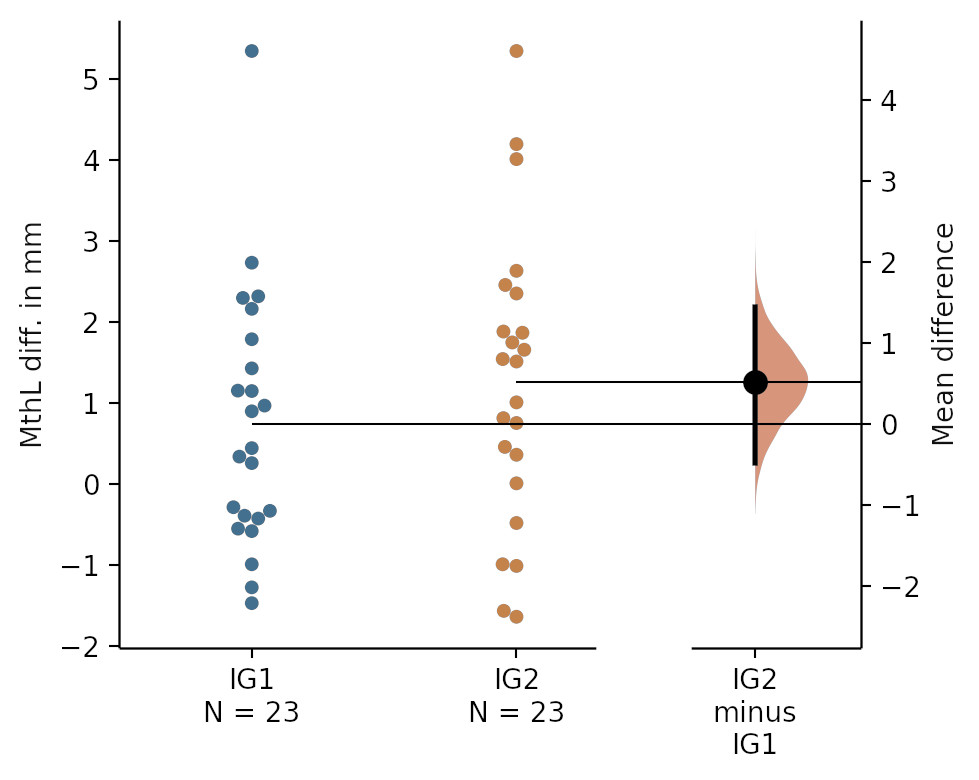

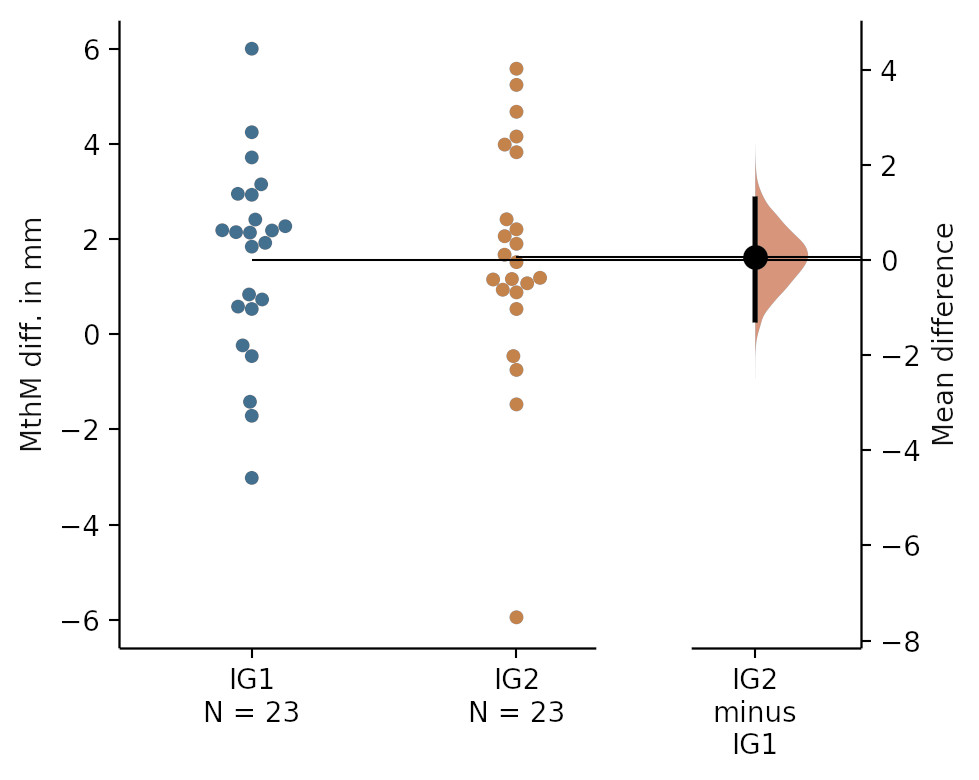


Figure F Comparison of progressions in muscle thickness in the stretching group (IG1) and the hypertrophy training group (IG2) in lateral head of the gastrocnemius and the medial head of the gastrocnemius illustrating the group differences in mean differences
